# Pharmacokinetic and Environmental Risk Assessment of Prime-2-CoV, a Non-Replicating Orf Virus-Based Vaccine against SARS-CoV-2

**DOI:** 10.3390/vaccines12050492

**Published:** 2024-05-02

**Authors:** Carina Metz, Verena Haug, Melanie Müller, Ralf Amann

**Affiliations:** 1 Institute of Immunology, University Hospital Tübingen, 72076 Tübingen, Germany; carina.metz@uni-tuebingen.de (C.M.); verena.haug@ifiz.uni-tuebingen.de (V.H.); melanie.mueller@uni-tuebingen.de (M.M.); 2 Institute for Tropical Medicine, Travel Medicine, and Human Parasitology, University Hospital Tübingen, 72076 Tübingen, Germany

**Keywords:** Orf virus, poxvirus, viral vector, vaccine, environmental risk assessment, biodistribution, shedding

## Abstract

Viral vector vaccines represent a substantial advancement in immunization technology, offering numerous benefits over traditional vaccine modalities. The Orf virus (ORFV) strain D1701-VrV is a particularly promising candidate for vaccine development due to its distinctive attributes, such as a good safety profile, the ability to elicit both humoral and cellular immunity, and its favorable genetic and thermal stability. Despite ORFV’s theoretical safety advantages, such as its narrow host range and limited systemic spread post-inoculation, a critical gap persists between these theoretical benefits and the empirical evidence regarding its in vivo safety profile. This discrepancy underscores the need for comprehensive preclinical validations to bridge this knowledge gap, especially considering ORFV’s use in humans. Our research introduces Prime-2-CoV, an innovative ORFV-based vaccine candidate against COVID-19, designed to elicit a robust immune response by expressing SARS-CoV-2 Nucleocapsid and Spike proteins. Currently under clinical trials, Prime-2-CoV marks the inaugural application of ORFV in human subjects. Addressing the aforementioned safety concerns, our extensive preclinical evaluation, including an environmental risk assessment (ERA) and detailed pharmacokinetic studies in rats and immunocompromised NOG mice, demonstrates Prime-2-CoV’s favorable pharmacokinetic profile, negligible environmental impact, and minimal ERA risks. These findings not only affirm the vaccine’s safety and efficacy but also pioneer the use of ORFV-based therapeutics, highlighting its potential for wider therapeutic applications.

## 1. Introduction

The development and application of viral vector-based vaccines represent a transformative approach in the fight against infectious diseases and cancer, offering new avenues for therapeutic intervention [1,2]. Among these, the attenuated Orf virus (ORFV) strain D1701-VrV, a member of the Parapoxvirus genus, has emerged as a particularly promising candidate for vaccine development. This is attributed to its unique combination of characteristics that distinguish it from other approved viral vectors currently in use. Notably, ORFV exhibits a very narrow host range, primarily affecting sheep and goats [3,4], which minimizes the risk of zoonotic transmission to humans. Furthermore, it is characterized by the absence of systemic virus spread post-inoculation, a feature that significantly enhances its safety profile for use in vaccines [5,6].

One of the most compelling advantages of ORFV-based vaccines is their capacity for effective re-immunization. This is due to the transient nature of vector-specific immunity elicited by the virus, which does not lead to the formation of long-lasting ORFV-specific neutralizing antibodies [7,8]. Consequently, this allows for repeated administration of the vaccine without diminishing its efficacy [9]. Additionally, ORFV-based vaccines have demonstrated potent immune-modulating properties, capable of inducing strong and durable immune responses against vector-encoded foreign antigens [10,11,12,13]. This is further augmented by the virus’s ability to stably integrate multiple transgenes within a single vectored vaccine, thereby enhancing its potential to target a wide range of pathogens and tumors [14].

Capitalizing on these advantageous properties, our team has developed Prime-2-CoV, an innovative ORFV-based vaccine candidate designed to combat COVID-19. Prime-2-CoV is engineered to express both the Nucleocapsid and Spike proteins of SARS-CoV-2, aiming to elicit a comprehensive immune response against the virus [15]. Formulated as a suspension for intramuscular (IM) administration, it is intended for active immunization to prevent COVID-19, caused by SARS-CoV-2. Currently undergoing clinical trials, Prime-2-CoV represents the first instance of ORFV being administered to humans, marking a significant milestone in the exploration of ORFV-based therapeutics.

Despite the theoretical safety advantages attributed to ORFV, there exists a gap in the empirical evidence supporting its safety profile, particularly concerning its behavior in vivo. Moreover, given that ORFV is employed as a recombinant viral vector and thus classified as a genetically modified organism (GMO), it necessitates a thorough environmental risk assessment (ERA). Such an assessment is imperative not only to evaluate potential implications for human health and the environment but also to ensure compliance with regulatory standards [16,17,18]. To bridge this gap in knowledge, our preclinical validation of Prime-2-CoV encompassed an ERA in conjunction with detailed pharmacokinetic studies post-IM and intravenous (IV) administration in rats. We extended our investigations to include pharmacokinetic analyses in immunocompromised NOG mice, examining the distribution and persistence of ORFV across various organs over time. We also examined the potential shedding of ORFV from vaccinated animals to assess any risk of environmental dissemination.

Our evaluation of the ERA for Prime-2-CoV was meticulously conducted, considering the unique attributes and the historical safety record of the ORFV vector used. This included a viral safety risk assessment and a detailed consideration of the properties of the incorporated transgenes. Through our ERA, we explored various factors pertinent to viral vector-based therapeutics [16,19]. Based on this comprehensive analysis, we deduced that the ORFV utilized for Prime-2-CoV presents an extremely favorable ERA profile, with risks assessed to be negligible [20]. Consequently, we deem no further risk mitigation strategies necessary.

In conclusion, the extensive preclinical investigations conducted on Prime-2-CoV affirm a positive pharmacokinetic profile, favorable biodistribution, and minimal shedding traits, all reinforcing the absence of systemic virus spread, clinical signs of illness, nor replication or shedding of the vector as well as a favorable ERA profile. These encouraging results lay a robust foundation for the continued clinical advancement of Prime-2-CoV, highlighting its potential as a safe and effective ORFV-based vaccine against COVID-19.

## 2. Materials and Methods

### 2.1. Virus and Cells

Based on the parental ORFV D1701-VrV-GFP-D12-Cherry, the full gene sequence of the Nucleocapsid protein and a modified Spike protein of SARS-CoV-2 were integrated through homologous recombination, resulting in the generation of D1701-VrV-CoV-Spike-D7-CoV-N, also known as Prime-2-CoV. The detailed generation of Prime-2-CoV is described in Reguzova et al. [15]. The recombinant virus D1701-VrV-GFP [21], which expresses the GFP protein, was used as a control.

VERO cells were cultivated in DMEM (Gibco, Life Technologies, Waltham, MA, USA), supplemented with 10% FCS (Capricorn Scientific, Ebsdorfergrund, Germany) and 1% PenStrep (Merck, Darmstadt, Germany) according to previous protocols [21]. Viral Production Cells (Invitrogen, Waltham, MA, USA) were cultivated in VPC medium (Gibco, Life Technologies, Waltham, MA, USA) as previously described [22]. 293T-ACE2 cells were cultured in DMEM (Gibco, Life Technologies, Waltham, MA, USA) supplemented with 10% FCS (Capricorn Scientific, Ebsdorfergrund, Germany), 2% L-Glutamin (200 mM, Gibco, Life Technologies, Waltham, MA, USA), 1% PenStrep (Merck, Darmstadt, Germany), 1% MEM non-essential Amino Acids Solution (100X, Gibco, Life Technologies, Waltham, MA, USA), and 1% Sodium Pyruvate (100 mM, Gibco, Life Technologies, Waltham, MA, USA), as described elsewhere [23].

### 2.2. In Vivo Experiments

All animal experiments were conducted under GLP conditions at BSL BIOSERVICE Scientific Laboratories Munich GmbH in compliance with German animal protection law. The studies have been subjected to Ethical Review Process and were authorized by the Bavarian animal welfare administration (#ROB-55.2-2532.Vet_03-18-63, #ROB-55.2-2532.Vet_03-17-38, #ROB-55.2-2532.Vet_03-18-63).

#### 2.2.1. Biodistribution Study in Wistar Rats

A total of 60 healthy Wistar rats, comprising an equal number of males and females aged 8–9 weeks (sourced from Charles River, Sulzfeld, Germany), were randomly divided into two groups, each consisting of 30 animals. These groups received a single dose of 1 × 10^8^ PFU of Prime-2-CoV, corresponding to the anticipated full human dose, administered either IM or IV, and afterward observed for 4 h, 24 h, 3 days, 5 days, or 8 days. During the entire observation period, animals were checked daily for clinical symptoms (central nervous system (abnormal behavior, posture, stereotypies), musculoskeletal system, skin and fur, eyes/mucous membranes, respiratory/pulmonary tract, cardiovascular system, digestive tract, reproductive tract, urinary tract), and morbidity and mortality were recorded. The body weight of all rats was measured once before the assignment to the experimental groups, on the first day of administration, and on the day of sacrifice. At the end of their respective observation periods, 6 animals per group (3 male and 3 female) were sacrificed using anesthesia (ketamine/xylazin) and subjected to necropsy. From each rat, lung, brain, heart, liver, spleen, both kidneys, ovaries for females or testes for males, muscle or tail (injection site), and mesenteric lymph nodes were collected and stored at ≤−70 °C for qPCR analysis. The heart was cut in two and one half was preserved in 4% neutral-buffered formaldehyde for histopathological examination. Additionally, blood was collected in EDTA-coated tubes and also stored at ≤−70 °C for qPCR analysis. For shedding analysis, urine and feces were sampled from all animals overnight in metabolic cages before their sacrifice at 24 h, 3 days, 5 days, and 8 days. Animals with necropsy at the timepoint 4 h were placed in metabolic cages directly after administration of Prime-2-CoV. If not enough feces could be obtained, it was collected as part of the sacrifice. Furthermore, one cotton swap per animal was moistened with saliva prior to necropsy and stored in tubes.

#### 2.2.2. Virulence Study in Mice

Healthy immunodeficient NOG (NOD.Cg-Prkdc^scid^ Il2rg^tm1Sug^/JicTac) (Taconic Biosciences A/S, Lille Skensved, Denmark) and immunocompetent NOD/ShiLtJ mice (strain 613) (Charles River, Sulzfeld, Germany), both female and aged 8–9 weeks, were administered either IM or IV with a single dose of 1 × 10^8^ PFU of Prime-2-CoV and observed for 3, 8, or 14 days. During the entire observation period of up to 14 days, animals were checked daily for clinical symptoms (see Section 2.5.1) and morbidity and mortality were recorded. Prior to administration of the vaccine, 4 h after vaccination, as well as on days 3, 8, and 14, rectal body temperature of the mice was determined. Additionally, the body weight of all animals was recorded once before the assignment to the experimental groups, on the day of vaccine administration, and on days 3, 5, 8, 11, and 14 after vaccination. On days 3, 8, and 14, 5 animals per group were sacrificed following anesthesia (ketamine/xylazin) and subjected to necropsy. Tissue from injection site (muscle or tail), lung, liver, spleen, and lymph nodes (located along the aorta between liver and bladder, pooled with popliteal lymph nodes) was collected and immediately frozen to determine Prime-2-CoV via qPCR analysis. One half of the heart from all animals was frozen as well, the other half was preserved in 4% neutral-buffered formaldehyde for histopathological examination.

#### 2.2.3. Shedding in New Zealand White Rabbits

Eighteen male and eighteen female healthy New Zealand White Rabbits (Charles River, Sulzfeld, Germany) were equally allocated to two groups, which received either three IM or three IV administrations of 1 × 10^8^ PFU or 3 × 10^7^ PFU of Prime-2-CoV, respectively, with a 2-week interval (days 0, 14, and 28). IM injection took place into the musculus biceps femoris of the right leg and IV injection into the right ear vein. During the entire observation period, animals were checked daily for clinical symptoms, and morbidity and mortality were recorded. On study days 1, 15, and 29, feces was collected in tubes, one cotton swap was moistened with saliva, and urine was collected directly out of the drip pan. Samples were stored at ≤−70 °C until qPCR analysis.

### 2.3. Histopathology

Histopathology evaluation of heart samples from mice and rats was carried out at AnaPath Services GmbH (Oberbuchsiten, Switzerland). Samples from all animals were trimmed, processed, embedded, cut at an approximate thickness of 2–4 micrometers, and stained with hematoxylin and eosin. Then, the slides were examined under light microscope by the study pathologist.

### 2.4. qPCR Analysis for Detection and Quantification of Prime-2-CoV in Tissues and Biofluids

A TaqMan^®^ PCR (qPCR) based assay was applied for detection and quantification of Prime-2-CoV in tissues and biofluids. The primers and probes were designed to cover a Prime-2-CoV specific nucleotide sequence region, which is expected not to be present in naturally occurring viruses or specimens. Since calibration standards of Prime-2-CoV were included, a signal-to-concentration plot can be generated, enabling the direct amplicon quantification of Prime-2-CoV. The whole qPCR analysis was carried out at Accelero Bioanalytics GmbH (Berlin, Germany).

#### 2.4.1. Sample Preparation

Lysis of tissue, biofluidic samples, and quality control samples along with DNA extraction had to be performed for subsequent qPCR analysis. Extraction quality control samples (EQCs) were prepared as positive and negative controls for each DNA extraction plate. EQC POS consists of 15 mg mouse/rat liver spiked with Prime-2-CoV while EQC NEG consists of 200 µL TE.

##### Lysis/Homogenization of Tissue Samples

Tissue samples from rats and mice were thawed and up to 30 mg (±10%) depending on the type of tissue (see Table 1) were transferred to homogenization tubes filled with ceramic beads. Briefly, 300 µL TE (Sigma-Aldrich, Taufkirchen, Germany) was added to the homogenization tubes in order to adjust tissue concentrations of up to 100 mg/mL, according to Table 1. Then, 300 µL of T1/Proteinase K Mix (25 µL Proteinase K (Macherey Nagel, Dueren, Germany) was added to 175 µL T1 (Macherey Nagel)), and tissues were then homogenized using the Precellys Evolution^®^ homogenizer (Bertin Technologies, Montigny-le-Bretonneux, France). The homogenization was performed at 6000 rpm and RT for 2 cycles (20 s each) with 30 s pauses between the cycles. Tissue samples were then lysed in a thermal mixer set at 56 °C and 500 rpm for 1 h. Subsequently, tissue samples were homogenized again at 6000 rpm and RT for 2 cycles (20 s each) with 30 s pauses between the cycles. For quality control purposes, EQC POS was treated the same way as tissue samples. For calibration standards, quality control samples, and EQC NEG samples, 200 µL of the respective solutions were mixed with 200 µL T1/Proteinase K Mix and then lysed in a thermal mixer set to 56 °C and 500 rpm for 1 h.

##### Lysis of Biofluid Samples

Samples of urine were thawed and mixed with 100 µL TE. For saliva swab samples, 200 µL TE was added. Then, urine and saliva samples were mixed with 200 µL T1/Proteinase K Mix and lysed at 56 °C and 500 rpm for 1 h. For rat blood samples, 100 µL of each sample was mixed with 100 µL TE. Then, 200 µL BQ1 and 25 µL Proteinase K were added, and lyses took place at 30 °C and 500 rpm for 20 min.

##### DNA Extraction

The DNA extraction was performed using NucleoSpin^®^ 96 Core Tissue Kit (Macherey Nagel) in accordance with the manufacturer’s instructions. Briefly, 400 µL Ethanol and 400 µL BQ1 were added to 400 µL of the lysed/homogenized sample. Then, samples were transferred to the NucleoSpin Tissue Binding Plate and centrifuged with 2210× *g*. After two washing steps, DNA was eluted in 60 µL BE buffer. The DNA extraction from blood samples was performed using the NucleoSpin^®^ 96 Blood Kit (Macherey Nagel) in accordance with the manufacturer’s instructions. Therefore, 200 µL ethanol was added to 200 µL lysed blood before adding the samples to the NucleoSpin Blood Binding Plate and, in the end, DNA was eluted in 60 µL elution buffer BE. DNA extraction monitoring of the samples was performed using a PCR assay specific for mouse or rat genomic DNA.

#### 2.4.2. qPCR Analysis

The ViiA™ 7 Dx Real-Time PCR System (Applied Biosystems, Carlsbad, CA, USA) was used for the analysis of all samples. For Prime-2-CoV-, rat-, and mouse-specific PCR, the respective PCR master mix was prepared (see Table 2), and 7 µL of the master mix was transferred to the respective well of a 384-well qPCR plate. The reaction mix for a single PCR reaction contained:

- 5 µL GoTaq Probe qPCR Master Mix (Promega, Madison, WI, USA) (with 60 nM CXR Reference Dye);
- 0.5 µL DNase-free water;
- 0.5 µL 18 µM forward primer (ACC-275 for Prime-2-CoV specific PCR, ACC-28 for mouse-specific PCR, ACC-186 for rat specific PCR);
- 0.5 µL 18 µM reverse primer (ACC-277 FOR Prime-2-CoV specific PCR, ACC-29 for mouse-specific PCR, ACC-187 for rat specific PCR);
- 0.5 µL 5 µM probe (ACC-276 for Prime-2-CoV specific PCR, ACC-30 for mouse-specific PCR, ACC-188 for rat specific PCR).

Then, 3 µL DNA extraction was transferred into the corresponding qPCR plate well and mixed with the 7 µL PCR master mix to result in a reaction volume of 10 µL. The analysis was performed in technical triplicates for the Prime-2-CoV-specific PCR and single determination for mouse- or rat-specific PCR. After sealing of the plate with sealing foil and a short centrifugation, the plate was transferred to the thermal cycling block. The PCR cycling program started at 95 °C for 3 min followed by 45 cycles of 95 °C for 15 s and 60 °C for 1 min.

#### 2.4.3. Calibration Standards

Seven calibration standards (2 × 10^2^, 4 × 10^2^, 1 × 10^3^, 2.5 × 10^3^, 5 × 10^3^, 2 × 10^4^, 2 × 10^5^ PFU per sample volume) were prepared to produce samples for assay calibration in the range of 2 × 10^2^ PFU (LLOQ) to 2 × 10^5^ PFU (ULOQ) in 200 µL TE. The lower limit of detection (LOD) of the assay is 2.5 × 10^1^ PFU per sample volume. The calibration standards were lysed according to section Lysis/Homogenization of Tissue Samples, and DNA was extracted according to section DNA Extraction, so that they were treated the same way as the in vivo study samples. Calibration standards were analyzed in triplicates in the qPCR reaction.

For calibration curve analysis, a linear regression model was applied using the least square method. The analyte amount (PFU) was plotted against the Ct values. The linear regression model is characterized by the following parameters: y-intercept, slope of the regression line, residual sum of squares (R^2^), PCR efficiency, and the formula: *Ct* = *slope* (log(*PFU*)) + *y* − *intercept*. The formula was used to back-calculate the PFU number (PFU) of the calibration standards, quality control samples, and in vivo samples.

### 2.5. In Vitro Assessment of Prime-2-CoV

#### 2.5.1. Immunofluorescence Staining

A total of 40,000 VERO cells were grown in an 8-well chamber slide (ibidi treat, ibidi GmbH, Gräfelfing, Germany), infected with Prime-2-CoV, with an MOI of 1 for 20 h, and fixed in 4% (*w*/*v*) methanol-free formaldehyde (Thermo Scientific, Waltham, MA, USA) at room temperature for 15 min followed by washing three times with PBS. For intracellular staining, cells were permeabilized with 0.2% (*v*/*v*) TritonX-100 in PBS at room temperature for 5 min and then washed three times with PBS. After blocking in 5% (*v*/*v*) BSA in PBS, cells were incubated with the respective primary antibodies (SARS-CoV-2 Spike Neutralizing Antibody, Rabbit Mab, 1:800 (Sino Biological, Beijing, China), SARS-CoV-2 Nucleocapsid Antibody, Mouse Mab, 1:200 (Sino Biological, Beijing, China)) in PBS containing 1% (*v*/*v*) BSA overnight at 4 °C. Followed by washing three times with PBS containing 1% (*v*/*v*) BSA, cells were incubated with secondary dye-coupled antibodies (Goat anti-Rabbit IgG, AF555, 1:400 (Invitrogen, Waltham, MA, USA) and FITC anti-mouse IgG, 1:400 (Biolegend, San Diego, CA, USA)) in PBS containing 1% (*v*/*v*) BSA at room temperature for 1 h. Cells were then washed three times with PBS and the nucleus was stained using DAPI (NucBlue Live Cell Stain ReadyProbes reagent, Invitrogen (Invitrogen, Waltham, MA, USA)). After washing, the slides were embedded in Mounting Medium (ibidi GmbH, Gräfelfing, Germany), and fluorescence images were taken using a Zeiss LSM800 Confocal microscope (ZEISS Research Microscopy Solutions, Oberkochen, Germany) (63× magnification with z-stack acquisition); images were recorded using Zen Blue 3.0 Software (accessed on 17 March 2024).

#### 2.5.2. Flow Cytometry

To assess the cell tropism of Prime-2-CoV, 293T cells that do (293T-ACE2) or do not (293T) express the ACE2 receptor were infected with Prime-2-CoV or D1701-VrV-GFP, a recombinant ORFV-encoding GFP instead of S and N, with an MOI of 3 for 20 h. For staining, the cells were washed twice with PBS and stained with Zombie Aqua™ dye (BioLegend, San Diego, CA, USA). To determine the infection rate, cells were stained with anti-ORFV antibody V1H1-AF647 at a 1:100 dilution (in-house).

To assess the antigen stability of Prime-2-CoV, the recombinant was passaged consecutively ten times in VERO cells. New cells were transfected with various dilutions of the viral harvest from the preceding passage. Cells were harvested after 2–3 days, and wells displaying approximately 50% infection were selected for the next round of infections. Subsequently, 500,000 Vero cells seeded in a 6-well plate were infected with Prime-2-CoV lysates isolated from both the first and tenth passages at an MOI of 1. After 20 h of incubation, the infected cells were detached using TrypLE (Gibco, Life Technologies, Waltham, MA, USA).

For staining, cells were washed twice with PBS and stained with Zombie Aqua™ dye and SARS-CoV-2 Spike Neutralizing Antibody (Rabbit Mab) at a 1:800 dilution (Sino Biological, Beijing, China) for 30 min at 4 °C. Cells were washed twice with PBS and incubated with secondary antibody conjugated with fluorescence dye, Goat anti-Rabbit IgG (AF488, 1:400, Invitrogen, Waltham, MA USA). For intracellular staining, cells were fixed using Fixation & Permeabilization Solution (BD Biosciences, Franklin Lakes, NJ, USA), and stained with SARS-CoV-2 Nucleocapsid Antibody (Mouse Mab) at a 1:200 dilution (Sino Biological, Beijing, China), for 30 min at 4 °C. Cells were washed twice with PBS and incubated with secondary antibody conjugated with fluorescence dye, anti-Mouse IgG (PE, 1:400, Biolegend, San Diego, CA, USA) in PBS. To assess the infection rate, cells were stained with anti-ORFV antibody V1H1-AF647 at a 1:100 dilution (in-house). Samples were fixed using 1% PFA in PBS and measured with BD LSRFortessa (BD Biosciences, Heidelberg, Germany) and analyzed using the FlowJo software 10.8.1 (accessed on 24 March 2024, FlowJo, LLC, Ashland, AL, USA). Data were gated on forward-scatter (FSC-A versus SSC-A), single-side scatter (SSC-A versus SSC-H), and live cells (Zombie Aqua negative).

#### 2.5.3. Growth Curve Analysis

Vero cells were infected with lysate of Passage 1 and 10 of Prime-2-CoV using an MOI of 0.1. After 24 h, 48 h, 72 h, and 96 h, infected cells were harvested, and viral titer was determined by plaque titration as described elsewhere [21].

### 2.6. Statistical Analysis

Required calculations were performed using Microsoft Excel version 1808 (accessed on 10 June 2021, Microsoft 365 Apps for Business). A statistical assessment of the results of the body weight was performed by comparing values of immunocompromised animals with immunocompetent animals using either a parametric one-way ANOVA and a post hoc Dunnett Test or a non-parametric Kruskal–Wallis Test and a post hoc Dunn’s Test, based on the results of homogeneity and normality tests. These statistics were performed with Ascentos 1.3.4 software or GraphPad Prism V.6.01 software. Graphs were created using GraphPad Prism V.10.1.2 software.

## 3. Results

### 3.1. Biodistribution and Pharmacokinetic Profile of Prime-2-CoV

To assess the biodistribution of Prime-2-CoV, we administered a single 1 × 10^8^ PFU dose IM or IV to a cohort of 30 Wistar rats, comprising an equal number of males and females. Post-vaccination, the rats were monitored for clinical symptoms, and their body weight was tracked until the designated endpoints at 4 h, and 1, 3, 5, or 8 days post-administration. Tissues, including lung, brain, heart, liver, spleen, kidney, ovaries, testes, injection site, mesenteric lymph nodes, and blood, were harvested for quantification of Prime-2-CoV using qPCR targeting the viral vector DNA. Additionally, half of the heart from each rat underwent histopathological examination.

**Figure 1:**
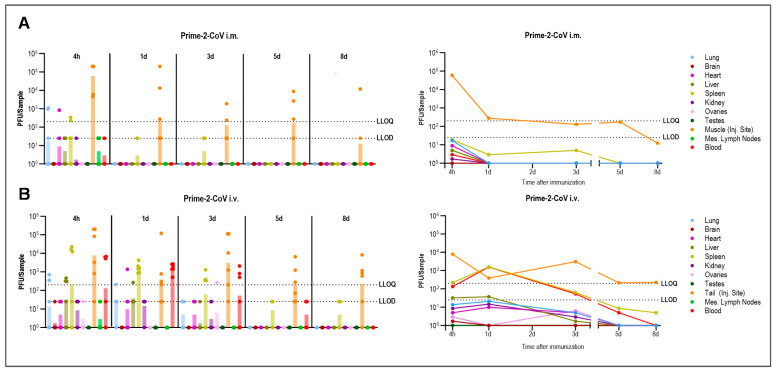
Biodistribution of Prime-2-CoV in Rats. Rats (*n* = 6 per timepoint) were immunized with Prime-2-CoV using 1 × 10^8^ PFU IM (**A**) or IV (**B**) and samples from lung, brain, heart, liver, spleen, kidney, ovaries, testes, muscle/tail, mesothelial lymph nodes, and blood were taken 4 h, 1 d, 3 d, 5 d, and 8 d later. Prime-2-CoV DNA abundance in isolated tissues was quantified by qPCR and calculated PFU are depicted. LLOD = Lower limit of detection, LLOQ = Lower limit of quantification. Samples with undetected Prime-2-CoV were assigned a value of 1.

Throughout the study, none of the animals succumbed, exhibited clinical symptoms, or presented with histopathological heart abnormalities post-Prime-2-CoV administration. Moreover, the body weight of the rats remained stable during the observation period. Although detectable levels of Prime-2-CoV (>25 PFU) were observed in various organs, quantifiable amounts (>200 PFU) were assessed in only 5% of all samples: at the injection site (15/29 animals), lung (2/30 animals), spleen (2/30 animals), and heart (1/30 animals), whereas for the latter three, this was only present at the 4 h mark post-vaccination (Figure 1A, left, Table 3). Beyond this time frame, Prime-2-CoV was predominantly present at the injection site, with a distinct reduction in virus levels over time (Figure 1A, right) and only a single animal showing quantifiable levels 8 days post-administration.

Conversely, IV administration resulted in generally higher and more sustained levels of Prime-2-CoV across most tissues, resulting in 14% of samples with quantifiable Prime-2-CoV levels. This was particularly evident in the blood and spleen, with quantifiable or detectable amounts also present in the lung, brain, heart, liver, kidneys, and ovaries (Figure 1B, right). Three days post-administration, Prime-2-CoV was detectable only in the spleen (4/6 animals), blood (4/6 animals), and site of administration (6/6 animals). Crucially, we observed a consistent and rapid decline in Prime-2-CoV levels across all tissues, with no indications of viral replication (Figure 1B, left), and at 8 days post-administration, Prime-2-CoV was only quantifiable at the site of administration. These data suggest that Prime-2-CoV exhibits a transient presence in rat tissues without propagation, aligning with the anticipated safety profile of the vaccine.

### 3.2. Evaluation of Prime-2-CoV Pharmacokinetics in Immunodeficient Animals

Given that Prime-2-CoV did not replicate in immunocompetent rats prompted us to explore whether its non-replicative nature would also be observed in immunodeficient animals, specifically NOG mice lacking B, T, and NK cells. Immunocompromised models are particularly suitable for assessing the replication capabilities of attenuated viruses due to their compromised ability to mount effective immune responses against infections [24]. Therefore, if Prime-2-CoV were to replicate, it would likely be evident in these immunodeficient mice. The absence of replication in this model would further affirm the vaccine’s attenuated nature and its safety for use in humans, especially for individuals with compromised immune systems due to conditions like genetic immunodeficiencies, ongoing chemotherapy, or HIV/AIDS, which face a heightened risk of severe infections.

To this end, we conducted a study involving 15 female NOG mice (NOD.Cg-Prkdc^scid^ Il2rg^tm1Sug^/JicTac) and 15 female NOD mice (NOD/ShiLtJ), the latter possessing a functional immune system. NOG and NOD mice are frequently used in preclinical experiments to assess the impact of the immune system on pharmacodynamics and pharmacokinetics. These models are well recognized and accepted by regulatory authorities for their relevance in immune system studies. Each mouse received a single 1 × 10^8^ PFU dose of Prime-2-CoV via IM or IV administration. Subsequently, the mice were monitored for clinical symptoms, and their body weight and temperature were recorded. The mice were then sacrificed at either 3, 8, or 14 days post-vaccination to collect lung, heart, liver, spleen, injection site, and lymph node tissues. Quantification of Prime-2-CoV in these tissues was performed using qPCR targeted at the viral vector DNA. Additionally, histopathological evaluations were conducted on one half of the heart.

**Figure 2:**
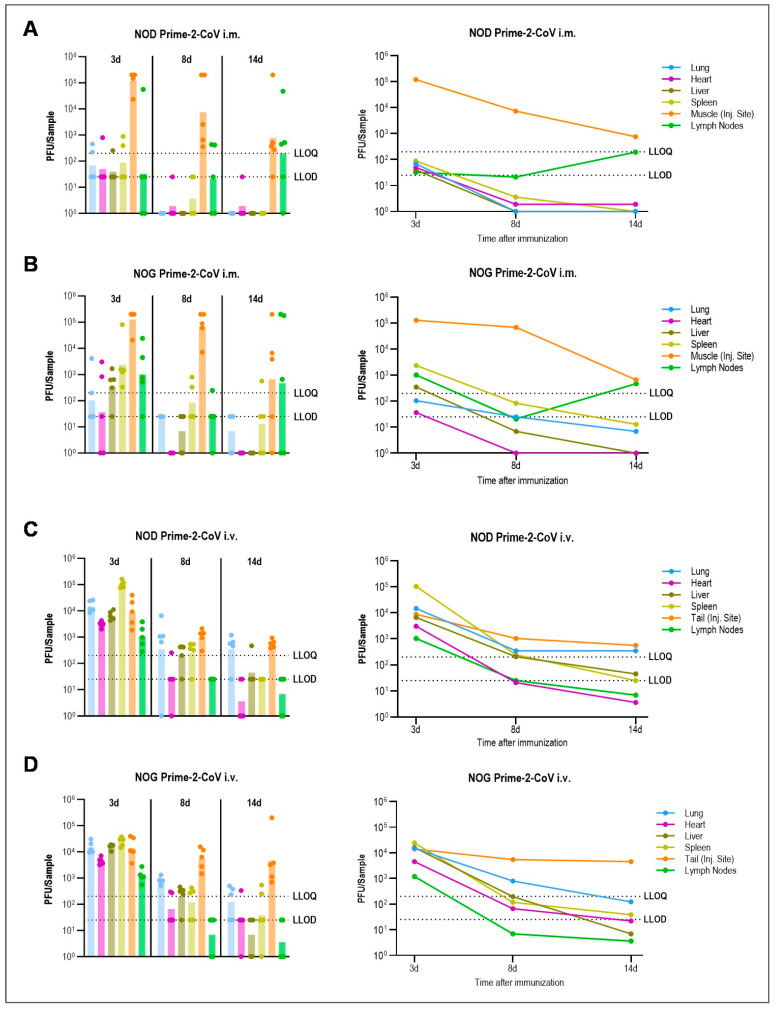
Biodistribution of Prime-2-CoV in NOG and NOD mice. Immunocompetent NOD mice (NOD/ShiLtJ) (**A**,**C**) and immunodeficient NOG mice (NOD.Cg-Prkdc^scid^ Il2rg^tm1Sug^/JicTac) (**B**,**D**) (*n* = 5 per timepoint) were immunized with Prime-2-CoV using 1 × 10^8^ PFU IM (upper box, **A**,**B**) or IV (lower box, **C**,**D**) and samples from lung, heart, liver, spleen, muscle/tail, and lymph nodes were taken 3 d, 8 d, and 14 d later. Prime-2-CoV DNA abundance in isolated tissues was quantified by qPCR and calculated PFU are depicted. LLOD = Lower limit of detection, LLOQ = Lower limit of quantification. Samples with undetected Prime-2-CoV were assigned a value of 1. Geometric mean for each timepoint is shown in the graphs on the right side.

In line with the results observed in rats, both the immunodeficient NOG and immunocompetent NOD mice did not exhibit any clinical signs of discomfort or evidence of heart lesions or inflammation post-administration of Prime-2-CoV. The animals maintained a normal body weight and temperature up to the point of sacrifice (Figure S1). Post-IM administration, the highest levels of Prime-2-CoV were detected at the injection site and lymph nodes. Notably, Prime-2-CoV was quantifiable in all sampled tissues at 3 days post-administration (Figure 2A,B, right). Despite this, the levels of Prime-2-CoV DNA declined over time in both mouse models, with lymph nodes being the exception (Figure 2A,B, left). Although clearance of Prime-2-CoV appeared notably slower in NOG mice, likely due to their compromised immune system, the downward trend in PFU values in tissues supports the virus’s inability to replicate.

After IV injection, higher levels of Prime-2-CoV were observed in the lung, heart, liver, and spleen, whereas lower levels were found at the injection site and in the lymph nodes, compared to IM injection (Figure 2C,D, right). While the presence of viral DNA at the tail vein injection site might initially seem surprising, it is important to emphasize that this likely resulted from minor inadvertent deposition outside the vascular pathway, a challenge common in small animal injections. Nevertheless, the viral copies detected at this site accounted for only about 0.01% of the injected dose, indicating that such occurrences have a minimal impact on the overall distribution of the vaccine. The consistent decline in Prime-2-CoV levels across all organs in both immunodeficient and immunocompetent mice, as well as the similar Prime-2-CoV DNA levels detected between the two groups, reaffirm the non-replicative nature of the virus, analogous to the IM injection results (Figure 2C,D, left). This further substantiates the safety profile of Prime-2-CoV in terms of its non-virulent behavior in both immunodeficient and normal immune system conditions.

### 3.3. Assessment of Shedding of Prime-2-CoV in Rats and Rabbits

**Figure 3:**
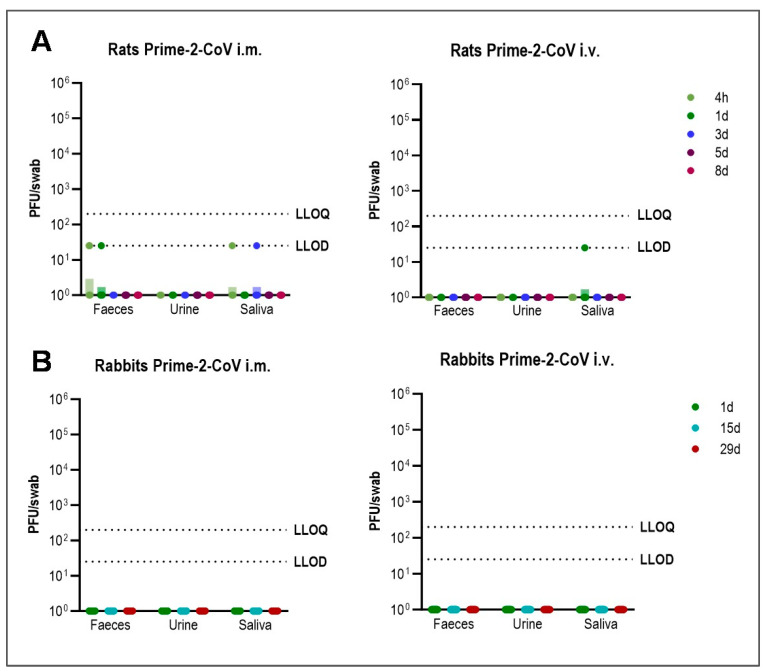
Shedding of Prime-2-CoV in Rats and Rabbits: (**A**) Rats (*n* = 6 per timepoint) were immunized with Prime-2-CoV using 1 × 10^8^ PFU IM (left) or IV (right) and samples from feces, urine, and saliva were taken 4 h, 1 d, 3 d, 5 d, and 8 d later. (**B**) Rabbits (*n* = 18 per timepoint) were immunized with Prime-2-CoV using 1 × 10^8^ PFU IM (left) or 3 × 10^7^ PFU IV (right) on days 0, 14, and 28, and samples from feces, urine, and saliva were taken on day 1, 15, and 29. Prime-2-CoV DNA abundance was quantified by qPCR and calculated PFU are depicted. LLOD = Lower limit of detection, LLOQ = Lower limit of quantification. Samples with undetected Prime-2-CoV were assigned a value of 1.

To evaluate the shedding potential of Prime-2-CoV, we conducted analyses on feces, urine, and saliva samples collected from vaccinated rats and rabbits. In rats, Prime-2-CoV was undetectable or present in trace amounts below the quantification limit, observed solely in select feces or saliva samples within the first three days following both IM and IV single administration in 5 out of 180 samples (Figure 3A). Given the sporadic nature of these detections without any consistent pattern, we consider these results as likely false positives, reinforcing the assay’s overall confidence level of 97.22%. For rabbits, which received vaccinations on days 0, 14, and 28 via either IM or IV routes, feces, urine, and saliva were collected for 24 h post-vaccination on each of these days and subsequently tested for Prime-2-CoV. Although high levels of SARS-CoV-2-specific antibodies were induced by day 28, no detectable levels of Prime-2-CoV DNA were found in any of the collected samples from rabbits, as illustrated in Figure 3B. These findings demonstrate a minimal risk of shedding, reinforcing the conclusion that Prime-2-CoV possesses a safe profile regarding environmental contamination following vaccination.

### 3.4. Environmental Risk Assessment of Prime-2-CoV

Our ERA was conducted in accordance with the methodology proposed by Baldo and colleagues [16]. This assessment evaluated potential risks associated with the ORFV backbone and the integrated transgenes, specifically the Spike and Nucleocapsid proteins of SARS-CoV-2, and finally for the resulting recombinant Prime-2-CoV, as summarized in Table 4. For the viral backbone, we considered factors such as intrinsic hazardous properties, the potential of insertional mutagenesis in the host genome, the risk of transmission of transmissible spongiform encephalopathies (TSE/BSE) and viral contaminants, and the potential for reversion to virulence (see Table 4, upper part). Regarding the risks posed by the integrated transgenes, we analyzed their inherent hazardous properties and conducted a literature review to evaluate the potential hazards associated with the Spike and Nucleocapsid proteins of SARS-CoV-2 (see Table 4, middle part). As these transgenes are incorporated into the Prime-2-CoV vaccine, we finally evaluated the risks and effects of the resulting recombinant Prime-2-CoV on host range, cellular tropism, potential effects on shedding, and the impact on viral replication efficiency (see Table 4, lower part).

#### 3.4.1. Risks Associated with the Parental Strain D1701-VrV

##### Origin of the Parental ORFV Vaccine Vector Strain D1701-VrV

The initial vaccine vector, D1701-VrV, that was used for the generation of Prime-2-CoV, originates from an ORFV: The ORFV strain D1701 was first isolated in 1972 from the mouth pustules of a lamb showing symptoms of the disease, and was subsequently named Düsseldorf No.1701. Through numerous passages in ovine and bovine cell cultures, the strain evolved into the highly attenuated ORFV D1701-B (with “B” indicating adaptation to Bovine cells), which proved harmless to lambs. By 1981, it received approval in Germany as a live vaccine against contagious ecthyma in sheep [25,26]. 

In 1998 and 1999, the D1701-B strain underwent adaptation to grow in VERO cell lines, which originate from the kidneys of African green monkeys. After 45 passages in VERO cell cultures, accompanied by a series of virus plaque purifications, a new, even more attenuated variant emerged, named D1701-V (with “V” indicating adaptation to VERO cells) [27]. Additionally, in 1999, the removal of the virulence gene vegf-e led to the development of the D1701-VrV strain (where “rV” denotes the removal of the vegf gene) [27,28]. This modification further reduced the virus’s virulence, making D1701-VrV the basis for generating new ORFV recombinants. Historically, the nomenclature for such recombinants has led to confusion, as recombinants have been referred to as “D1701-V-X, -Y, or -Z”, (where ‘-X, -Y, or -Z’ denotes any integrated transgene) despite the deletion of the vegf gene, making it challenging to differentiate these from the D1701-V virus that still includes the vegf gene. For clarity and improved understanding, it is proposed that all recombinants based on D1701-V and having the vegf-e gene deleted will henceforth be designated as “D1701-VrV-X, -Y, or -Z”.

Before being modified with specific transgenes for product development, the ORFV was subject to a stringent de-risking process to address concerns about TSE/BSE and viral contaminants. This process entailed four rounds of plaque purification in VERO cells from a certified Good Manufacturing Practice (cGMP)-banked Working Cell Bank (WCB), ultimately resulting in single plaque wells within a 384-well plate. The Vero cells, known for their resistance to TSE/BSE agents [29], facilitated a purification process that diluted the original viral material by over a factor of 10^34^. This significant dilution effectively minimized any associated risks of TSE/BSE and other viral contaminants, rendering them negligible.

##### Risk of Reconversion to Virulence

In the context of recombination or reversion to wild type, it is important to highlight that the ORFV strain D1701-VrV, which serves as the backbone for Prime-2-CoV, is significantly attenuated. This attenuation has led to the elimination of several virulence factors [14], resulting in a replication deficiency in vivo, even within immunocompromised hosts, without causing any signs of illness. Consequently, the likelihood of the virus reverting to a virulent form is substantially reduced. While theoretically, there might be a possibility of co-infection with Prime-2-CoV and a naturally occurring poxvirus, we regard this risk as negligible for a number of reasons.

Firstly, the method of administration for Prime-2-CoV is IM, in contrast to the natural ORFV infections, which typically enter the body through skin lesions, often at distinct locations. Moreover, parapoxvirus infections in humans are exceedingly rare, with minimal reports, indicating the absence of circulating parapoxviruses in humans that could enable recombination events. Additionally, no human-to-human transmission of these viruses has been documented. Finally, the removal of multiple virulence factors from Prime-2-CoV further decreases the likelihood of reversion to virulence, as it would necessitate the unlikely event of simultaneously restoring several virulence factors. These factors collectively greatly reduce the likelihood of recombination events leading to reversion to a more virulent form.

##### Potential of Insertional Mutagenesis in the Host Genome

Poxviruses stand out among DNA viruses due to their exclusive replication within the host cell’s cytoplasm [30]. Similarly, ORFV, and by extension, Prime-2-CoV, are confined strictly to the cytoplasm, which significantly reduces worries related to their integration into the host genome. Consequently, the likelihood of genomic insertion leading to transformative changes in the host cells is considered negligible.

#### 3.4.2. Transgene Intrinsic Risk Evaluation

The Spike protein, which assembles into heterotrimers on the viral surface, facilitates entry into host cells. This process is initiated by the binding of the receptor-binding domain (RBD) within the S1 subunit to the host’s angiotensin-converting enzyme 2 (ACE2) receptor, followed by membrane fusion mediated by the S2 subunit [31]. 

In the design of Prime-2-CoV, we have engineered a modified version of the Spike protein intended to preserve the prefusion structure of the Spike protein, which is targeted by neutralizing antibodies and is critical for an effective immune response. This includes the incorporation of the D614G mutation, which is associated with increased infectivity and transmission of the virus [32,33]. Additionally, we have introduced the K986P and V987P proline substitutions, which are known to stabilize the prefusion conformation of the Spike protein [34]. Furthermore, to prevent the conformational change that is necessary for viral fusion and entry into the host cell, we have deleted the furin cleavage site (converting amino acids RRAR to GSAS at positions 682 to 685) [34]. Despite its pivotal role in SARS-CoV-2 infectivity, the Spike protein does not exhibit intrinsic hazardous effects, as evidenced by several preclinical studies validating the immunogenicity and safety of recombinant viral vectors that express the Spike protein sequences [35,36,37,38,39].

The Nucleocapsid protein is recognized for its strong immunogenicity, stability, and conservation across coronavirus species, making it a viable antigen target for vaccines against SARS-CoV-2 and its variants. It binds to the viral RNA within the virion, aiding in RNA replication, virion assembly, and release. Within host cells, the Nucleocapsid protein has been implicated in cell cycle deregulation, interference with the interferon-mediated immune response, and apoptosis induction [40,41]. Despite these observations, which suggest a capacity for harm, the use of the Nucleocapsid protein in various vaccine strategies has not been linked to any adverse effects when expressing its sequences, underscoring its suitability in vaccine formulations [42,43].

#### 3.4.3. Risks Associated with Recombinant Prime-2-CoV Vector

##### Impact of Transgene Expression on Tissue Tropism

Poxviruses predominantly produce mature virions (MVs), along with enveloped variants. These include intracellular enveloped virions (IEVs), which can evolve into extracellular enveloped virions (EEVs) by acquiring an additional outer membrane derived from the host cell plasma membrane during release. Consequently, EEVs may display host cell proteins, including surface proteins, on their envelope [44,45]. 

The Spike protein is crucial for cross-species transmission and significantly influences host tropism [46], underscoring its relevance in ERA. This context stresses the importance of monitoring the Spike protein’s expression in Prime-2-CoV.

**Figure 4:**
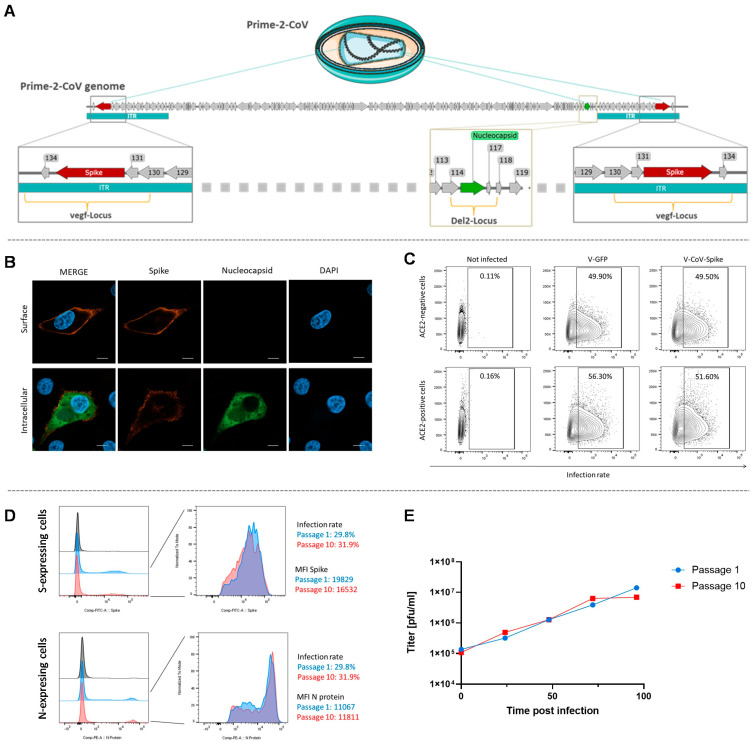
In vitro assessment of Prime-2-CoV: (**A**) Illustration of Prime-2-CoV. Arrows indicate the open reading frames within the viral genome of the D1701-VrV-based Prime-2-CoV vaccine. The inverted terminal repeats (ITRs) are featured in turquoise at both the left and right ends of the genome. The transgene for the SARS-CoV-2 Spike protein, depicted in red, is integrated into the vegf-Locus, which is situated in the ITR. The transgene for the SARS-CoV-2 Nucleocapsid protein, shown in green, is inserted in the Del2-Locus. (**B**) Vero cells were infected with Prime-2-CoV. Surface staining and Intracellular staining was performed using anti-Spike and anti-Nucleocapsid antibodies, respectively. While the Spike protein is expressed on the surface, the Nucleocapsid protein was detected intracellularly. Scale bar = 10 µm. (**C**) 293T cells that do (293T-ACE2) or do not (293T) co-express the ACE2 receptor have been infected with Prime-2-CoV or a recombinant ORFV encoding an irrelevant antigen (GFP, D1701-VrV-GFP). ORFV-infected cells are positive for the ORFV-specific Ab V1H1. The frequency of cells infected with ORFV was measured by flow cytometry. No increased susceptibly of ACE2+ cells were detected indicating no altered susceptibility of Prime-2-CoV. (**D**) Prime-2-CoV was passaged 10 times in Vero cells. For antibody staining, Vero cells were infected with Passage 1 and 10 for 20 h. Infected cells were stained with anti-ORFV antibody V1H1-AF647 and anti-Spike antibody. After permeabilization, cells were stained with anti-Nucleocapsid protein antibody. (**E**) Vero cells were infected with Passage 1 and Passage 10 of Prime-2-CoV (MOI 0.1). After 24 h, 48 h, 72 h, and 96 h, samples were taken and the viral titer was determined.

In our in vitro investigations, the Spike protein was detected on the surface of infected cells (Figure 4B), prompting an examination of possible changes in cell tropism. We compared the infectivity of Prime-2-CoV on ACE2-expressing HEK cells against a control ORFV vector that expresses GFP rather than the Spike protein. Our results indicated no noticeable difference in the rate of infection between Prime-2-CoV and the ORFV-GFP control (as indicated in Figure 4C). Furthermore, when examining Prime-2-CoV through electron microscopy using gold-labeled anti-SARS-CoV-2 Spike antibodies, we did not detect the Spike protein on the surface of Prime-2-CoV particles. 

Moreover, our biodistribution studies did not indicate a preference of Prime-2-CoV for tissues expressing ACE2, e.g., lungs, kidney, heart, or liver. Although the ACE2 receptors of rats and mice are structurally analogous to those of humans, their binding efficiency and susceptibility to natural SARS-CoV-2 Spike protein interaction are notably diminished. Additionally, the unclear mechanisms of Orf virus cell entry and unidentified entry receptors present challenges in projecting these findings directly to humans. 

While we have not found evidence of altered host tropism, the possibility cannot be completely ruled out. Considering that Prime-2-CoV does not replicate in vivo and is administered IM at controlled doses, the potential for inducing harmful adverse events is deemed low. Moreover, our studies have shown no signs of viral shedding, leading us to conclude that the transgenes do not influence either shedding or viral replication. Therefore, we suggest that the incorporation of transgenes into Prime-2-CoV does not adversely affect the vaccine’s risk profile.

##### Genomic Stability of Prime-2-CoV

The Spike and Nucleocapsid antigens of Prime-2-CoV, being of large and medium size, respectively, are inherently prone to potential deletion through the process of repeated passaging. The stability of these transgenes within the ORFV genome is imperative, not only for the vaccine’s effectiveness against SARS-CoV-2 but also to ensure uniform expression through the various stages of scaling up production. Moreover, maintaining the genetic stability of the vector itself is critical; any variations could potentially affect its vaccine characteristics and attributes. From a quality control perspective, it is essential that the virus retains its replication efficiency and transgene expression capabilities consistently. To evaluate these aspects, we conducted a comprehensive analysis focusing on the genetic stability and transgene expression of the S and N antigens in Prime-2-CoV over 10 passages. This study also looked into potential shifts in the virus’s growth characteristics throughout these passages. Our research verified the stable integration of the transgenes within the ORFV genome, with no mutations detected. As a result, the expression of both antigens remained constant up to the 10th passage, without any change in the level of expression (see Figure 4D). Additionally, sequencing revealed no genomic alterations over the course of the passages. The growth characteristics of Prime-2-CoV also remained consistent (Figure 4E), reinforcing the vaccine’s stability and its suitability for mass production.

## 4. Discussion

Prime-2-CoV, a multi-antigenic SARS-CoV-2 vaccine utilizing the ORFV strain D17101-VrV, presents theoretical safety benefits. However, empirical evidence for its safety, especially regarding in vivo performance, has been lacking. As we transition Prime-2-CoV from laboratory research to clinical trials, we conducted an extensive examination of its pharmacokinetic characteristics and its safety for the environment. This research provides crucial insights into the viability of ORFV-based vaccine technologies. Being the first ORFV-based therapeutic to enter clinical trials, Prime-2-CoV represents a significant breakthrough in the development of ORFV-based therapeutics. Our research confirms the pharmacokinetic benefits and safety profile of Prime-2-CoV, while also highlighting its low environmental impact. These findings position D1701-VrV-based vaccines as a promising option for future vaccination efforts.

Ensuring the highest degree of safety is a fundamental requirement for vaccines. Therefore, the inability of vectors to replicate in vivo is a critical feature, as vectors lacking replication capacity are considered to be markedly safer [47]. This characteristic allows for a more refined management of the immune response compared to replication-competent vectors and significantly reduces the risk of transmitting the vaccine vector to non-immunized individuals. The benefits of such safety and control are generally considered to outweigh the potential disadvantages, such as the need for higher dosages or the possibility of eliciting weaker immune responses [2]. Consequently, a significant number of authorized vaccines have been engineered to be non-replicating, with prominent examples being those based on human adenovirus serotypes, such as serotype 5 in CanSino’s vaccine and serotype 26 in Janssen’s vaccine. Similarly, the ChAdOx1 vaccine by AstraZeneca and the University of Oxford uses the non-replicating Chimpanzee adenovirus ChAdY25. This non-replication feature is achieved by deleting the E1 gene, which is crucial for viral DNA replication and transcription of viral genes, thus hindering the vector’s replication capability but not its ability to deliver genes to host cells. To manufacture these E1-deficient vectors, specialized packaging cell lines that provide the missing E1 function are required [48]. However, there is a theoretical risk that during vector production, homologous recombination events could lead to the generation of replication-competent adenoviruses (RCAs) due to the presence of E1 sequences in the packaging cell line. These events could potentially allow the vector to regain its replication ability [48]. Additionally, there is a hypothetical risk that RCAs could form post-vaccination through recombination events between the vector and any wild-type viruses infecting the same cell. Yet, to date, there have been no reported cases for the latter, suggesting that this risk may be more theoretical than practical. Moreover, Modified Vaccinia Ankara (MVA), utilized in the development of the approved Ebola vaccine MVA-BN-Filo, is characterized by its non-replication in mammalian hosts [49]. MVA is a highly attenuated orthopoxvirus, adapted to avian cells through 570 serial passages in chicken embryo fibroblasts, resulting in a 15% loss of its genome. This adaptation significantly reduced its virulence and pathogenicity, and minimizes the risk of reverting to a more virulent form [50]. Although there is a theoretical risk that deleted genes could be restored through recombination with naturally occurring orthopoxviruses during co-infection [51], this is deemed highly unlikely due to the absence of suitable human poxviruses for such recombination.

ORFV offers significant advantages for vaccine development, such as strong induction of humoral and cellular immune response and the potential for multiple re-administrations without anti-vector immunity interference. Its ability to incorporate multiple antigens into a single vector and excellent thermal stability also enhance its suitability for human vaccines. However, to date, no studies have specifically explored the biodistribution and pharmacokinetics of the ORFV virus in general, and D1701-VrV in particular. Our findings reveal that following IM administration in rats, Prime-2-CoV was initially detectable in a modest fraction of samples, with notably low levels of the virus observed, except at the site of administration. Importantly, ORFV levels declined continuously in all examined organs, becoming undetectable in locations other than the administration site within five days post-administration. This pattern underscores the vaccine’s non-replicating nature and its temporary presence within the body. Alongside IM administration, we also investigated the biodistribution and pharmacokinetic characteristics of Prime-2-CoV after IV administration. This evaluation aimed to analyze these factors under exacerbated conditions, thereby refining our understanding and substantiating the potential risks more robustly. It is important to note that IV administration is not the preferred method for prophylactic vaccination against infectious diseases due to its impracticality. As expected, we found differences between IM and IV administration routes, with the latter typically resulting in higher and more prolonged levels of the vaccine in various tissues. These findings were supported by studies in immunodeficient NOG mice, known for their compromised immune systems. Despite observing generally higher viral titers initially, a steady decrease in ORFV levels in all examined organs was documented with lymph nodes being the exception. Notably, the observed increases in DNA within the lymph nodes following intramuscular administration likely reflect the natural transport of vaccine components from the injection site to lymphatic tissues, a common immunological response where lymph nodes act as reservoirs for antigens. This phenomenon could explain the elevated DNA levels detected. Moreover, the consistent decline in DNA levels over time in mice immunized intravenously supports the absence of active viral replication. Importantly, these observations are in line with previous studies showing no replication of ORFV in peripheral blood mononuclear cells, which share many cell types with lymph nodes [52]. This consistency across different studies suggests a non-replicative nature of our ORFV vector in vivo. These findings are particularly relevant for the development of safe vaccines for individuals with compromised immune systems, such as those with genetic immunodeficiencies, undergoing chemotherapy, or living with HIV/AIDS. Our findings offer an in-depth examination of the biodistribution and pharmacokinetics of D1701-VrV, demonstrating a clear safety profile characterized by the non-replicating nature of Prime-2-CoV and its rapid elimination. These results not only broaden our understanding of the vaccine’s behavior in vivo but also highlight its potential applicability to various vaccination strategies, including those aimed at immunocompromised patients. Such insights could be particularly valuable in the development of therapeutic vaccination strategies where IV administration of immunocompromised individuals might be advantageous [53].

Shedding refers to the release of the vaccine virus into the environment through excretions such as feces, saliva, or other bodily fluids. Shedding is generally rare and typically associated with live attenuated vaccines rather than those utilizing non-replicating viral vectors. However, because shedding can present several potential concerns, its evaluation is considered beneficial during both the preclinical and clinical stages of vaccine development [54]. In our studies, we detected no measurable levels of Prime-2-CoV in the urine, feces, or saliva of rabbits or rats, even after IV vaccination. Given that the rats received a full human dose, yet weigh approximately 1/200th of an adult human, this significantly bolsters the vaccine’s safety profile. Our findings align with data from adenovirus (Ad)- or Modified Vaccinia Ankara (MVA)-based vaccines, where shedding post-intramuscular administration is extremely rare or has not been reported [16,55]. Consequently, we assess the risk of shedding with our vaccine as negligible.

In addition to evaluating biodistribution, pharmacokinetics, and shedding, we conducted our ERA in accordance with the methodology proposed by Baldo and colleagues [16]. Likewise, we assessed the potential risks associated with the parental ORFV strain D1701-VrV that was used for the generation of Prime-2-CoV, the transgenes Spike and Nucleocapsid of SARS-CoV-2 that were integrated into Prime-2-CoV, and of the resulting recombinant Prime-2-CoV vaccine, too. Our evaluation is summarized in Table 2 and made use of four distinct methodologies:

1. Qualitative Risk Assessment (QRA), which applies subjective judgment to evaluate risks based on their potential impact and the likelihood of occurrence, rather than concrete evidence. 2. Literature Review-based Risk Assessment (LitRA), which involves a thorough analysis of existing scientific literature, including prior studies on similar vaccines, to compile evidence regarding safety and efficacy. 3. Historical Data Review-based Risk Assessment (HisRA) that scrutinizes available historical data, to anticipate potential risks and outcomes. 4. Experimental Risk Assessment (ExpRA), which employs data obtained from structured experimental setups to assess risks based on empirical evidence.

The parental strain D1701-VrV has undergone the loss of several virulence factors, leading to its significant attenuation, and the chances of recombination events arising from co-infection with the vector are extremely low. Collectively, this results in a very low risk of the virus reverting to a virulent form. Furthermore, like all poxviruses, D1701-VrV replicates solely in the cytoplasm, eliminating the risk of genomic integration into the recipient’s DNA. Additionally, the risk of transmitting TSE/BSE is minimized. Consequently, we determine that the risk associated with using D1701-VrV for the generation of recombinant vaccines is considered negligible.

Furthermore, we have conducted a thorough review of the existing literature to evaluate the risk associated with the SARS-CoV-2 transgenes integrated into Prime-2-CoV. For the Spike protein, the evidence base is more robust, as most vaccine strategies have focused on targeting this protein, indicating that the associated risk is very low. In contrast, there are fewer approaches targeting the Nucleocapsid protein, and most of these involve concurrent targeting of the Spike protein [35,36,42,56]. While there is a lack of extensive evidence, there are currently no indications of any risk associated with the Nucleocapsid protein in vaccine formulations. Thus, we assess the risk related to the transgenes as low.

Finally, we assessed the risks associated with the generated recombinant Prime-2-CoV. We were able to demonstrate genomic stability, effective transgene expression, and replication efficiency across 10 passages, exceeding the requirements for manufacturing this drug. This stability offers an advantage over vectors with smaller genomes, which might struggle with larger transgene loads [2]. Our evaluation of cellular tropism revealed no significant changes in cell targeting due to the Spike or Nucleocapsid proteins, even in ACE-2-expressing cells. Therefore, we concluded, that Prime-2-CoV does not pose a higher risk than its parental strain, D1701-VrV.

While our study on Prime-2-CoV provides promising insights into its pharmacokinetic profile, safety, and environmental impact, it is important to acknowledge certain limitations. Firstly, the scope of our pharmacokinetic analysis was confined to small animal models, including rats and immunocompromised NOG mice. While these models are invaluable for initial assessments, they do not fully replicate human physiology. Therefore, the extrapolation of these results to human subjects should be approached with caution, and further validation in human clinical trials is needed [57]. Secondly, while the study did not find evidence of viral replication in vivo, which supports the vaccine’s safety, the absence of viral replication in immunodeficient mice models does not unequivocally guarantee similar outcomes in all human populations. Immunological variances across individuals and populations may influence the vaccine’s behavior and efficacy. Moreover, our environmental risk assessment, while thorough, might not cover all possible environmental scenarios, particularly under unexpected conditions of viral release. Finally, the study’s duration did not allow for the observation of long-term effects, including potential delayed adverse reactions. Most importantly, the transition from preclinical findings to clinical application necessitates careful consideration. Therefore, future studies need to address these limitations through expanded clinical trials, which are crucial for thoroughly evaluating the vaccine’s efficacy, safety, and long-term effects on a diverse human population.

## 5. Conclusions

The findings from our study affirm the pharmacokinetic advantages and the favorable safety profile of Prime-2-CoV, also emphasizing its minimal impact on the environment. Given that this comprehensive preclinical assessment represents the inaugural exploration into an ORFV-based therapeutic, its implications are particularly noteworthy. With Prime-2-CoV marking the first application of ORFV in human subjects, the significance of this research extends beyond its immediate results, laying a foundational pathway for the continued exploration and advancement of ORFV-based therapeutic solutions.

## Figures and Tables

**Table 1:** Tissue concentration for Lysis/Homogenization.

Tissue	Concentration for Lysis/Homogenization [mg/mL ± 10 %]	Tissue Amount [mg ± 10%]
Lung	100	30
Brain	100	30
Heart	100	30
Liver	50	15
Spleen	25	7.5
Kidney	100	30
Ovaries	40	12
Testes	100	30
Injection site (muscle or tail)	50	15
Mesenteric lymph nodes	35	10.5
Feces	50	15

**Table 2:** Primer and Probe sequences.

Reagent	Sequence (5′→ 3′)
Prime-2-CoV specific	
Forward primer ACC-275	GCGGCGTATTCTTCTCGGAC
Reverse primer ACC-277	TCGATGCGGTGCAGCAC
Probe ACC-276	FAM-TGCGGTAGAAGCC-MGBEQ
Rat specific	
Forward primer ACC-186	TTGGAAGGTGAAGTGTGGTCTT
Reverse primer ACC-187	AGCTCAACCTGCTTCCTCTCTAT
Probe ACC-188	FAM-TTCAGCCTTCTGGAGAGGAGCC-BHQ1
Mouse specific	
Forward primer ACC-28	TACCTGCAGCTGTACGCCAC
Reverse primer ACC-29	GCCAGGAGAATGAGGTGGTC
Probe ACC-30	TAMRA-CCTGCTGCTTATCGTGGCTG-BHQ2

FAM = 6-Carboxyfluorescein, BHQ1 = Black hole quencher 1, MGBEQ = Minor groove binder eclipse quencher, TAMRA = Tetramethylrhodamine.

**Table 3:** Biodistribution in rats following a single dose of Prime-2-CoV. This table summarizes data from all samples across all timepoints evaluated.

Tissue/Biofluid	Administration Route: IM		Administration Route: IV	
	No. of Samples Analyses	No. of Prime-2-CoV Positive (>200 Calculated PFU)	No. of Samples Analyzed	No. of Prime-2-CoV Positive (>200 Calculated PFU)
Blood	30	0	30	12
Brain	30	0	30	0
Faeces	30	0	30	0
Heart	30	1	30	1
Kidney	30	0	30	0
Liver	30	0	30	4
Lung	30	2	30	3
Mesenteric lymph nodes	30	0	30	0
Injection site	29	15	30	21
Ovaries	15	0	15	0
Saliva	30	0	29	0
Spleen	30	2	30	13
Testes	15	0	15	0
Urine	29*	0	29	0
Total:	388	20	388	54

* One sample delivered a quantifiable result but was excluded from evaluation as no rat-specific DNA could be detected.

**Table 4:** Summary of Risk Assessments for Prime-2-CoV.

Potential Risk	Measure	Assessed by	Risk Evaluation
**Risks Associated with the Parental Strain D1701-VrV Used for the Generation of Prime-2-CoV**			
Level of attenuation and the related risk of reversion to virulence	i. Detailed assessment of the history of parental virus strain including genomic alterations (e.g. loss of virulence factors) ii. Evaluation of pathogenicity in (immunocompromised) hosts	HisRA	Negligible
Integration of ORFV sequences in recipient’s genome	i. Consideration of poxvirus-specific characteristic to replicate exclusively in cytoplasm, but not in nucleus. ii. Consultation of topic-related literature	LitRA + QRA	Negligible
Risks of transmission of TSE/BSE and other viral contaminants	De-Risking process performed to dilute initial material by the factor 10^34^	QRA	Negligible
**Risks Associated with integrated Transgenes**			
Intrinsic hazardous properties of the S and N antigens	Reviewing relevant literature to evaluate characteristics such as pathogenicity, toxicity, and oncogenic potential.	LitRA	Low
**Risks Associated with recombinant Prime-2-CoV vector**			
Lack of genetic stability and integrity of transgenes	Genomic stability and transgene expression assessment over 10 passages.	ExpRA	Low
Impact on host range, cellular or tissue tropism	i. Biodistribution and pharmacokinetic study in rats and mice after IM and IV administration. ii. In vitro studies with ACE-2 expressing cell lines.	ExpRA	Negligible
Impact on replication efficiency in vivo	Pharmacokinetic study in immunocompromised mice after IM and IV administration	ExpRA	Negligible
Recombination probability between Prime-2-CoV and naturally occurring homologs	i. Consideration of factors: parapoxvirus incidence, route of administration (IM) vs. natural infection (skin), likelihood for recombination resulting in more virulent offspring. ii. Consultation of topic-related literature	LitRA + QRA	Negligible
Spreading or dissemination of Prime-2-CoV to the environment	Assessment of Shedding of Prime-2-CoV in rats (IM and IV administration) and rabbits (IM administration)	ExpRA	Negligible

HisRA: Historical Data Review-based Risk Assessment, LitRA: Literature Review-based Risk Assessment, QRA: Qualitative Risk Assessment, ExpRA: Experimental Risk Assessment.

## Data Availability

The data that support the findings of this study are available from the corresponding author upon reasonable request.

## Supplementary Materials

The following supporting information can be downloaded at https://www.mdpi.com/article/10.3390/vaccines12050492/s1, Figure S1: Development of Body Weight in Rats (A) and NOD and NOG Mice (B) as well as Body Temperature in Mice (C) after Vaccination with Prime-2-CoV.
