# Supplementary material for: Pharmacokinetic and Environmental Risk Assessment of Prime-2-CoV, a Non-Replicating Orf Virus-Based Vaccine against SARS-CoV-2
Source: Vaccines (Basel). 2024 May 2;12(5):492. doi: 10.3390/vaccines12050492 (PMC11126055; doi:10.3390/vaccines12050492)
Supplement: Supplementary file 1 [file vaccines-12-00492-s001.zip › vaccines-2957190-supplementary.pdf]

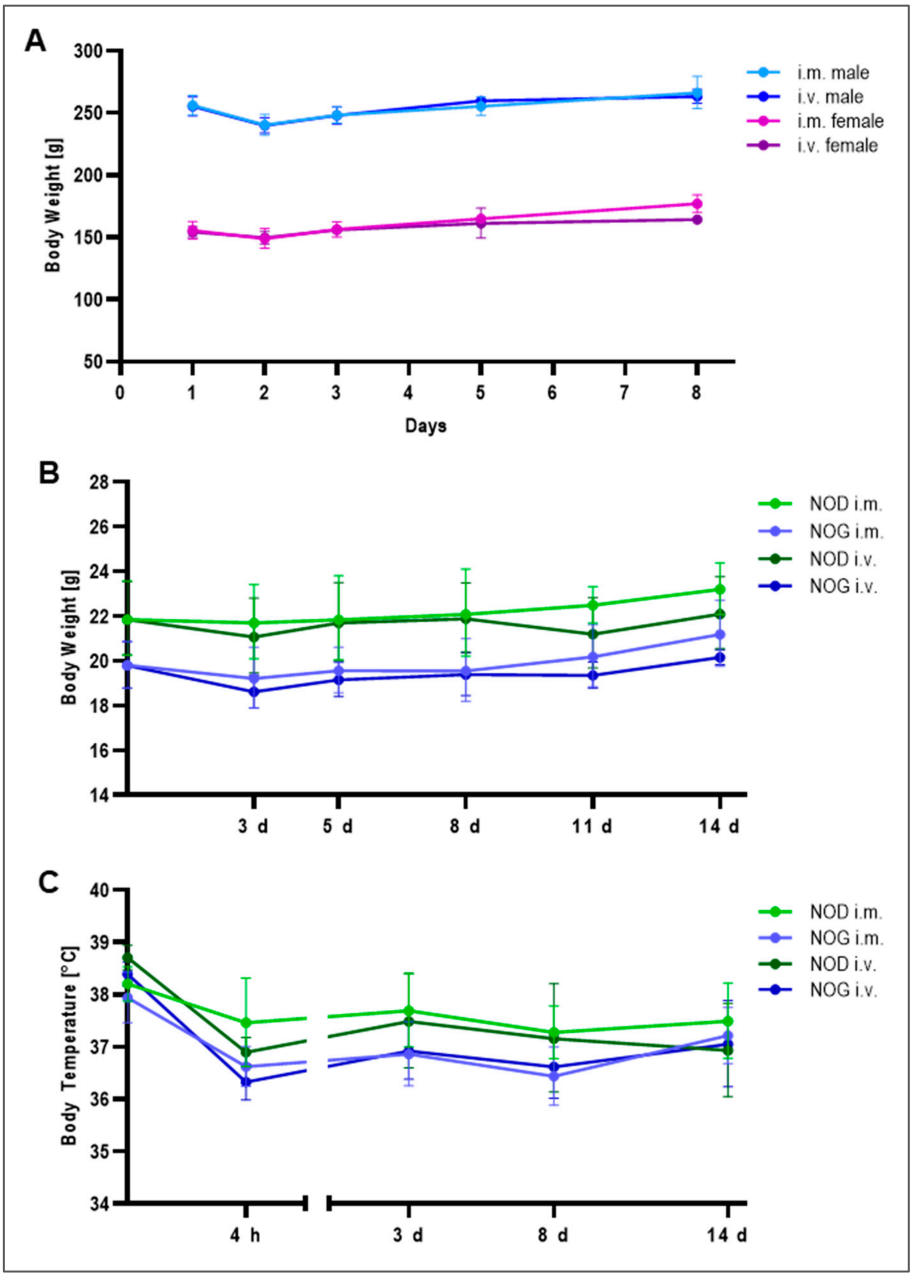

Supplement Figure S1: Development of Body Weight in Rats (A) and NOD and NOG Mice (B) as well as Body Temperature in Mice (C) after Vaccination with Prime-2-CoV.
